# Effects of Airborne Particulate Matter on Respiratory Health in a Community near a Cement Factory in Chilanga, Zambia: Results from a Panel Study

**DOI:** 10.3390/ijerph14111351

**Published:** 2017-11-06

**Authors:** Emmy Nkhama, Micky Ndhlovu, J. Timothy Dvonch, Mary Lynam, Graciela Mentz, Seter Siziya, Kuku Voyi

**Affiliations:** 1 Department of Environmental Health, Chainama College of Health Sciences, P.O. Box 33991, Lusaka 10101, Zambia; 2 Department of Clinical Medicine, Chainama College of Health Sciences, P.O. Box 33991, Lusaka 10101, Zambia; makobani@yahoo.com; 3 Department of Environmental Health Science, School of Public Health, University of Michigan, Ann Arbor, MI 48109, USA; dvonch@umich.edu (J.T.D.); lynam@umich.edu (M.L.); 4 Department of Health Behavior and Health Education, School of Public Health, University of Michigan, Ann Arbor, MI 48109, USA; gmentz@umich.edu; 5 Public Health Unit, School of Medicine, Copperbelt University, P.O. Box 71191, Ndola 10101, Zambia; ssiziya@gmail.com; 6 School of Health Sciences, University of Lusaka, P.O. Box 36711, Lusaka 10101, Zambia; 7 School of Public Health and Health System, Health Sciences Faculty, University of Pretoria, P.O. Box 667, Pretoria 0001, South Africa; kuku.voyi@gmail.com

**Keywords:** cement production, emissions, PM_2.5_, PM_10_, respiratory symptoms, lung function, community, Zambia

## Abstract

We conducted a panel study to investigate seasonal variations in concentrations of airborne PM_2.5_ and PM_10_ and the effects on respiratory health in a community near a cement factory; in Chilanga; Zambia. A panel of 63 and 55 participants aged 21 to 59 years from a community located at the edge of the factory within 1 km and a control community located 18 km from the factory respectively; were followed up for three climatic seasons July 2015 to February 2016. Symptom diary questionnaires were completed and lung function measurements taken daily for 14 days in each of the three climatic seasons. Simultaneously, PM_2.5_ and PM_10_ concentrations in ambient air were monitored at a fixed site for each community. Mean seasonal concentrations of PM_2.5_ and PM_10_ ranged from 2.39–24.93 μg/m^3^ and 7.03–68.28 μg/m^3^ respectively in the exposed compared to the control community 1.69–6.03 μg/m^3^ and 2.26–8.86 μg/m^3^. The incident rates of reported respiratory symptoms were higher in the exposed compared to the control community: 46.3 vs. 13.8 for cough; 41.2 vs. 9.6 for phlegm; 49.0 vs.12.5 for nose; and 13.9 vs. 3.9 for wheeze per 100 person-days. There was a lower performance on all lung indices in the exposed community compared to the control; overall the mean FEV1 (forced expiratory volume in one second) and FVC (forced vital capacity) predicted percentage for the exposed was six and four percentage points lower than the control. Restriction of industrial emissions coupled with on-going monitoring and regulatory enforcement are needed to ensure that PM (airborne particulate matter) levels in the ambient air are kept within recommended levels to safeguard the respiratory health of nearby community residents.

## 1. Introduction and Background

Worldwide, airborne particulate matter (PM) in outdoor ambient air has received increased attention due to its associations with cardiovascular and respiratory morbidity and mortality. The Global Burden of Disease Study ranked PM as the ninth leading risk factor for respiratory and cardiovascular diseases and various cancers [1]. Additionally, in the same year PM was ranked fifth on the list of top causes of all-cause mortality [2]. Globally approximately 3.2 million premature deaths are attributed to exposure to PM annually [3]. Particulate matter of aerodynamic diameter less than 2.5 microns (PM_2.5_) and less than 10 microns (PM_10_) are of public importance because they are respirable in size leading to pulmonary diseases. Both long term (reductions in lung functions) and short term (cough, wheeze, phlegm and shortness of breath) respiratory effects due to exposure to PM have been reported globally [4,5,6,7,8,9,10]. The most commonly reported respiratory symptoms include cough, wheeze, dyspnea, sneezing and phlegm [11,12,13,14]. Additionally, lung function measured as forced expiratory volume in one second (FEV1), forced vital capacity (FVC), ratio of forced expiratory volume in one second and forced vital capacity (ratio of FEV1/FVC), peak expiratory flow rate (PEFR) and peak expiratory flow (PEF) has also been shown to be reduced [10,12,15,16].

Even though thresholds have been identified, the adverse effect of exposure to PM concentrations below these thresholds has been observed. The World Health Organization (WHO) recommends exposure levels not exceeding 10 µg/m^3^ annually and 25 µg/m^3^ in 24-h mean (not exceeding for more than 3 days a year) for PM_2.5_; and 20 µg/m^3^ annually and 50 µg/m^3^ in 24-h mean for PM_10_ [17]. Notwithstanding the WHO recommendations, countries have established different cut-off levels as safe exposure [18]. The decision for these levels is determined mainly by economic considerations [19]. Developed countries have more stringent standards and advanced strategies to reduce air pollution with PM than developing countries [18]. For instance, the U.S. Environmental Protection Agency (EPA) air quality standard is set at 12 µg/m^3^ annual and 35 µg/m^3^ in 24-h concentration for PM_2.5_, and 150 µg/m^3^ in 24-h not to be exceeded more than once per year on average over a 3-year period for PM_10_. Emerging economies like China and India are beginning to build their environmental management systems and have set their standards as follows; 70 µg/m^3^ annual and 150 µg/m^3^ for 24-h for PM_10,_ and 75 µg/m^3^ in 24-h mean concentration for PM_2.5_ for urban areas [20]. Among countries in the southern African region only South Africa has set exposure level standards; for PM_10_ of 60 µg/m^3^ annual and 180 µg/m^3^ maximum in 24-h concentration [17]. Zambia, like several other countries that have not promulgated their own standards due to local constraints and capabilities, uses WHO air quality guidelines. 

The cement industry is a major contributor to total global PM emissions [19,21]. Within a cement factory, considerable amounts of PM as dust is generated at almost every stage of the manufacturing process; from quarrying of the raw material to the packing [22]. The PM result as fugitive dust within and in surrounding areas of cement plants. Cement dust derived PM levels above the minimum acceptable values have been reported in both the factory plant and communities residing near the cement plants. For instance, Tiwari et al. reported high levels of PM in a community located about 1.5 km from a cement factory that exceeded the WHO recommendations [23]. In a related study [24] in Nigeria, the total atmospheric dust was reported at an average concentration of 650 μg/m^3^, more than 600 μg/m^3^ higher than the recommended safe limit of 25 μg/m^3^ set by the Federal Environmental Protection Agency (FEPA). Similarly, other cement plant locations have found evidence of total PM concentration ranging from 196.19 μg/m^3^ to 423.83 μg/m^3^, which is above the 24-h average WHO guideline value of 120 μg/m^3^ for total PM concentration [22]. In a related study [25], one community lying within a radius of 1.2 km of a cement factory experienced PM_10_ concentrations higher than the recommended 24-h mean on more than the recommended maximum 35 days annually. Several other studies [8,26,27], that assessed air quality in similar communities, have demonstrated comparable findings.

Currently, most evidence in literature regarding the effect of the emissions from cement production is from studies conducted within the factory plants and involved mostly workers. Understanding the effects of exposure to cement dust on human respiratory health for communities residing near cement factories is imperative, as it would allow for interventions that would balance between cement production and protection of human health. This is possible only when knowledge about the extent of the air pollution and its adverse health effects is measured precisely. The objective of this study, using a prospective panel study, was to investigate the seasonal variations in concentrations of PM (PM_2.5_ and PM_10_) and effects on respiratory health in a community around a cement factory, in Chilanga, Zambia. 

## 2. Study Methodology

### 2.1. Study Site

The study site is a community called Freedom Compound, situated near one of Zambia’s major cement producing plant, Lafarge Chilanga Cement factory. The plant is located 15 km south of Lusaka, the capital city of Zambia, at geographical coordinates of 15.3875° S and 28.3228° E. Freedom compound is located right at the edge and within 1 km of the factory, on the north-western and leeward side of the plant. A community (Bauleni) situated 18 km north from the plant and from the study site, was included as control for comparison. The choice of the control community was dictated by the need to have a community with a socioeconomic profile as similar to Freedom Compound as possible but outside the fallout zone. Details of the study sites are given elsewhere [28].

### 2.2. Sampling of Study Participants

**Figure 1:**
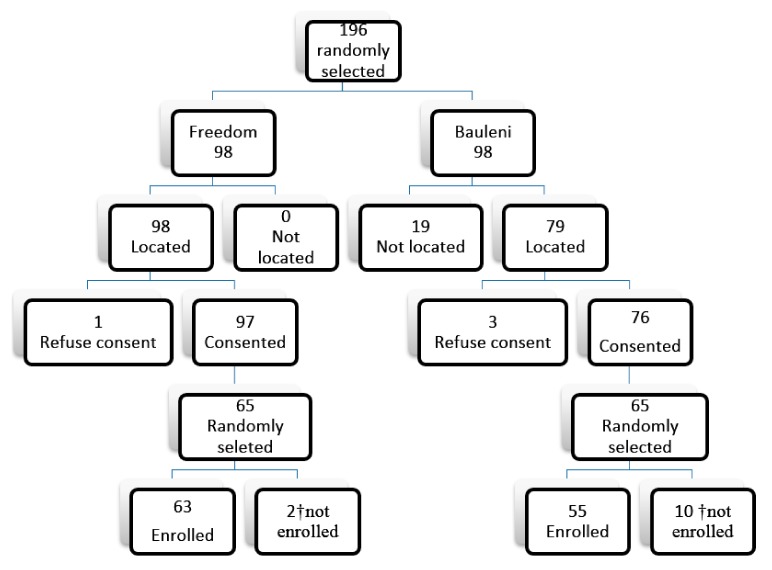
Schematic drawing of participant selection. ^†^ total of 12 were not enrolled; 4 changed residential place, 5 found employment outside study area consent and 3 withdrew consent.

Participants for the panel study were randomly selected as a sub-sample from a sampling frame compiled during a cross-sectional study conducted in the two communities over a year earlier. The study was reviewed and approved by a local research ethics committee in Zambia-ERES Converge IRB (00005948) and from IRBs of the Universities of Pretoria (0000 2535 IORG 0001662) and Michigan (00070842). Participant recruitment for the cross sectional study is given elsewhere [28]. For this study, based on a power analysis, a minimum sample size of 55 per community was required to give us power to test our research question of interest. As we assumed loss to follow-up of 30% and further that 50% of the participants from the cross-sectional study may refuse consent or that they may not be found for reasons such as relocation or death, having been recruited a year earlier, we inflated the minimum sample size by 80% giving us a total sample of 98 per community. As illustrated in Figure 1, we then randomly choose 98 potential participants from each sampling frame of each community followed by physically visiting the communities to locate the participant. We located 98 and 79 potential participants from Freedom and Bauleni respectively. We sought verbal consent from the located potential participants for them to take part in the panel study. In Freedom all, except one consented, while three refused consent in Bauleni. The consenting potential participants constituted a second tier sampling frame; 65 were drawn randomly from each community for enrollment to make a panel. However, because data collection commenced a month later after seeking their verbal consent, only 63 and 55 participants from Freedom and Bauleni respectively finally participated (enrolled). Eligibility criteria included: participants should have lived in the community for 24 months and were likely not to relocate within the next 18 months, aged between 21 and 59 years, consenting to be followed-up for the study period, not working for the cement factory or any construction company and spent 80% of the time in either Freedom or Bauleni.

### 2.3. Study Design

A prospective panel study was utilized; participants were followed-up over three climatic seasons. In each season (cold dry from 28 July–14 August 2015, hot dry from 15–28 October 2015, and warm rainy from 7–21 February 2016) participants were visited daily for 14 consecutive days. On these days, daily symptoms questionnaires were completed and spirometry done for each participant.

The first data collection wave comprised the enrolled 55 and 63 participants from Bauleni and Freedom communities respectively. In the second data collection wave, 51 and 57 participants were followed-up in Bauleni and Freedom respectively. Eight participants, four from each community, were lost to follow up. Of the four participants lost to follow up in Bauleni, three relocated while one found a new job opportunity outside the community; left early and got home very late. Similarly, one participant in Freedom had changed residence and three found jobs away from Freedom community. In the third data collection wave, there was no loss to follow up.

### 2.4. Data Collection

#### 2.4.1. Measurement of Covariates

The medical personnel that conducted the visit completed questionnaires and conducted spirometry measurements. At the beginning of each data collection wave for each season the gender, date of birth (DOB), height and weight were collected for each participant. Age was calculated as the difference between the DOB and the date at the commencement of the study, height and weight was measured to the nearest centimeter and kilogram (without shoes) using a stadiometer and a balance scale respectively. Smoking status was classified as “never” (reference group), “former” and “current”. Former smoker was any participant who ceased smoking at least two years before the first data collection wave, while a current smoker was any participant who used rolled or manufactured cigarette during the study or ceased smoking less than two years before the first data collection wave. Gender was coded as male and female. Cooking fuel was classified as “electricity” or “charcoal” while lighting fuel referred to “electricity” (torch or electric bulb) or “candle”. Location of the kitchen in relation to sleeping space was measured as “inside” (cooking and sleeping done in the same room) or “outside” (kitchen separate from sleeping space). In multivariate analyses never smoked, use of electricity for lighting and cooking, male and cooking outside the house were used as reference.

#### 2.4.2. Air Monitoring

To assess daily and seasonal variability of airborne PM concentrations in the two communities, ambient air monitoring was conducted during the three measurement waves reflecting the three climatic seasons. A community-level monitoring station was set up in each of the two communities. The equipment was placed on a building rooftop at each site allowing sampling inlets to be 2–3 m off the ground and away from any interference to air circulation. The choice of the two sites was based on security considerations for the monitoring equipment. Filter-based measurements of PM_2.5_ and PM_10_ were made daily during each seasonal exposure assessment field intensive (every two weeks in duration) at each sampling location. All PM samples were collected daily, over 24-h durations. Measurements were made using 2-μm pore, 47-mm Polytetrafluorethylene (PTFE) Teflon membrane filters (Pall, Ann Arbor, MI, USA) [29]. Vacuum pump systems were used to draw air through the sample at a nominal flow rate of 16.7 L/min using Teflon-coated aluminum cyclone inlets (University Research Glassware, Chapel Hill, NC, USA) [4]. Flow determinations were made at the beginning and end of each sampling period using a calibrated rotameter (Matheson Inc., Montgomeryville, PA, USA) [4]. For this method, analytical precision was calculated to be within 10% based on replicate analysis with a limit of detection of 5.1 μg (calculated as three times the standard deviation of seven repeated blank filter measurements). All measurements were above the detection limit.

All filters collected were prepared and analyzed at the University of Michigan Air Quality Laboratory (UMAQL) [4]. All gravimetric determinations of Teflon filters were made using a microbalance (Mettler MT-5; Mettler Toledo, Columbus, OH, USA) in a temperature/humidity-controlled Class 100 clean laboratory and followed the Federal Reference Method [4], which included conditioning filters for 24 h in the clean lab. At the conclusion of each study period, collected filters were shipped back to the UMAQL, conditioned for 24 h, and post-weighed following the same protocol used for filter pre-weight. For this method, analytical precision was calculated to be within 10% based on replicate analysis, with a limit of detection of 5.1 μg (calculated as three times the standard deviation of seven repeated blank filter measurements). All measurements were above the detection limit.

#### 2.4.3. Measurement of Incidence of Respiratory Symptoms

A daily symptom diary questionnaire that collected information on daily respiratory symptoms was administered to each participant. Respiratory symptoms referred to acute respiratory symptoms; cough, shortness of breath, wheezing, runny nose, breathlessness and sneezing. The daily diary symptom instrument was adapted from previous survey instruments [30,31]. The diary questionnaires were completed for each participant daily by the medical personnel that conducted the spirometry. This was necessary to maintain consistency as some of the participants were not able to read and/or write; and to maintain compliance to the study protocol and for quality control.

#### 2.4.4. Measurement of Lung Functions

Lung function testing was performed using the EasyOne ultrasonic flow-sensing spirometer (NDD Medical Technologies, Zurich, Switzerland) [32]. Before taking measurements, the procedure was explained and demonstrated to each participant at the beginning and during each data collection period until the participant was comfortable to perform the maneuver on their own without any assistance. Standard methods were used to determine the validity and reproducibility of the blows.

Lung function was measured as FEV1, FVC and FEV1/FVC ratio. For daily session, performed between 07:30 a.m. and 09:30 a.m., the participant had to blow into the EasyOne spirometer. The maneuver involved the participants inspiring fully, seal the nose with one hand and place the mouth around the mouthpiece of the spirometer, followed by breathing out as fast as they could until the lungs were empty. The measurements were carried out according to guidelines and techniques for performing spirometry according to American Thoracic Society (ATS) standards. The quality of spirometry tests was assessed according to the ATS guidelines [30].

#### 2.4.5. Measurements of Meteorological Characteristics

The meteorological data used in this study were obtained from the Zambia Meteorological department (ZMD). These included the daily minimum and maximum temperatures (°C), minimum and maximum relative humidity (%), minimum and maximum wind speeds (m/sec), minimum and maximum rainfall (in mm), and hours of sunshine (solar radiation) for the months July/August and October 2015; and February 2016. The wind direction is predominantly east-west most of the year with light variable northerlies and north-easterlies during the rainy season. We calculated the daily average temperature (using daily minimum and maximum temperatures). We also calculated the daily average relative humidity and reported the average of the two.

### 2.5. Statistical Analysis

Descriptive statistics were used to describe the baseline sample characteristics. For time invariant characteristics, means (and their SD (standard deviation)) for continuous normally distributed variables or medians (and the IQR (interquartile range)) for non-normally distributed were calculated while proportions and 95% CI were determined for categorical variables. Baseline (or cross-sectional) comparison for statistical differences of these characteristics between the exposed and control community were tested with student *t*-test (or Mann Whitney; an equivalent non-parametric test) and the chi-Square test for continuous and categorical variables respectively. For time variant covariates model based generalized estimating equations (GEE) comparisons of longitudinal means were carried out. A *p*-value of <0.05 was considered as statistically significant. Meteorological data was analysed as 24-h mean temperatures and humidity. For air quality monitoring 24-h mean concentrations of PM_2.5_ and PM_10_ were determined. 

We used person-day follow-up period to determine the incidence rate (per 100 person-days) of respiratory symptoms in the two communities. The total person-day follow-up time was the sum of the days that each participant was followed-up and completed the symptom diary. Each symptom’s incidence was the sum of the number of days that each participant reported a particular symptom. Lung function indices were analysed as percentage of predicted values except for FEV1/FVC ratio where absolute values were used.

Associations between the PM with respiratory symptoms and lung functions were estimated by fitting different multivariate generalized estimating equations (GEE) regression models for each outcome; binomial and Gaussian distribution were assumed for categorical and numeric outcome variables respectively. To account for auto-correlation in outcomes for each panel, we specified exchangeable correlation matrix. Furthermore, potential confounders were adjusted for: sex, age, height, weight, smoking history, socioeconomic status, asthma and meteorological variables. We also tested for lag effect of the exposure on the outcomes for days 1–7. Modification effects for age and gender were investigated with the inclusion of interaction terms. Quasi-likelihood information criterion (qic) was used to select the fit of the models. STATA version 12 (Stata Corp L 2011, College Station, TX, USA) was used for all analyses.

## 3. Results

Table 1 provides a description of the cohort study population. We recruited 116 participants; 63 and 53 from Freedom and Bauleni communities respectively with 4753 person-days data points. An 87.1% completion rate was achieved. Loss to follow-up was mostly due to relocation of study participants from the respective study sites. On average 52 participants from both communities completed the daily symptom diary symptom for each season. The average age for participants was 30 and 40 years from the exposed and control group respectively. There was no statistically significant difference in age, weight, smoking status, employment status of the study participants, source of energy used for cooking and lighting between the two communities (*p* value > 0.05). Very low proportion of participants reported being current smokers; 4.8% and 5.5% for Freedom and Bauleni, respectively. Electricity was the most commonly used source of energy for lighting and cooking in both communities. Generally, the prevalence of all the respiratory symptoms were higher in the exposed than control community and the difference was statistically significant (*p* value < 0.05).

Table 2 gives a summary of 24-h PM concentrations (PM_2.5_ and PM_10_) in the ambient and meteorological parameters across the seasons from cold dry through to wet season by location. The PM_2.5_ concentrations were higher in the exposed compared to control community. Further seasonal variations were observed in both communities; the highest concentrations were recorded in the hot dry season compared to the other two seasons. For the exposed community, the seasonal 24-h mean concentrations (in μg/m^3^) ranged from 2.39 in warm rainy to 24.93 in hot dry season whereas the 24-h mean concentrations for the control community were significantly lower ranging from 1.9 μg/m^3^ in warm rainy season to 6.89 μg/m^3^ in hot season. The 24-h mean concentrations for PM_10_ for both communities tended to follow the same pattern as that of PM_2.5_. In the exposed community, concentrations ranged from 7.03 μg/m^3^ in warm rainy to 68.28 μg/m^3^ in hot season and from 2.26 μg/m^3^ warm rain to 8.82 μg/m^3^ hot dry season. There was no statistically significant difference in meteorological characteristics (temperature and humidity) between the exposed and control sites during the study period (*p* value = 0.557 and 0.658, respectively).

Cough and nose irritation were the commonly reported symptoms. Additionally, the incidence rates of the symptoms were higher in Freedom compared to the control (Table 3). Furthermore, incidence rate of the symptoms showed variation from season to season within each community. However, the variation was wider in the exposed compared to the control (e.g., the incidence rate of cough ranged was 12.4% and 15.9% in Bauleni while 10.4% and 75.1% in Freedom for the warm rainy and hot dry seasons respectively). Similar observations were made of the other symptoms. To be noted also is that the lowest incidence rate for each of the symptoms regardless of community was observed in the warm rainy season and highest in the hot dry season. The transitional probabilities, i.e., the chance of a participant reporting a particular symptom during follow-up given they did not report having the symptom at the beginning, were several folds higher in Freedom compared to the control. For instance, the chance of reporting nose irritation and cough was 10 and 4 times higher respectively in Freedom compared to the Bauleni.

The percentage of the predicted values of the lung function indices of participants is presented in Table 4. Overall, the mean percentage of the predicted FEV1 and FVC was lower in the exposed compared to the control by 6% and 4% respectively. The indices showed seasonal variations in both communities; lowest in the hot dry season and highest in the cold dry (89.67 vs. 94.0 for FEV1; and 91.13 vs. 94.61 for FVC). Besides, being lower for Freedom, the percentage of the predicted lung function showed also minimal variation compared to the control community. The FEV1 ranged from 89.7–91.6 for Freedom and 96.5–99.4 for Bauleni while FVC ranged from 91.1–94.2 for Freedom and 94.6–100.0 for Bauleni. The spirometric airflow limitation (FEV1/FVC ratio) was lower for the exposed group compared to the control (0.82 vs. 0.84). A FEV1/FVC ratio of less than 80%, which is the accepted cut threshold, was observed in 32.1% of the exposed participants and 11.5% in the control.

Generally, PM_2.5_ was a significant predictor for occurrence of the respiratory symptoms expect for wheeze; a 1 µg/m^3^ increase in PM_2.5_ increased the odds of cough, phlegm and nose irritation by about 2% controlling for season, smoking status and asthma (Table 5). However, it had an opposite effect on the odds of wheeze (*p* value < 0.05). Overall, an increase in PM_10_ concentration reduced the odds of all the symptoms, but was only statistically significant for phlegm and nose irritation. Daily assessment of PM_10_ showed a statistically significant effect for phlegm and nose irritation 3–5 days after exposure (lag 3 and 5); phlegm lag 5 [OR = 1.00 (0.06–1.00)]; and nose irritation lag 3 [OR = 1.00 (1.00–1.10)] and [OR = 1.00 (0.06–1.01)].

Table 6 shows results from single-pollutant models. Although not statistically significant, a 10 µg/m^3^ increase of delayed exposure in PM_2.5_ was found to decrease FEV1 by 72.0 mL and 157 mL in the exposed and control respectively. There was an 82 mL decrease in FVC for a 10 µg/m^3^ increase in PM_2.5_, however this was not statistically significant. A lag effect, although not statistically significant, was seen in the exposure to PM_10_; for a 10 µg/m^3^ increase in PM_10_ FEV1 decreased ranging from 60 mL to 154 mL with the highest decline observed three days after exposure (lag 3). Similarly, FVC showed a decline ranging from 60 mL to 262 mL on day 5 (lag 5), PM_10_ marginally statistically reduced FVC by 262 mL in the exposed community.

## 4. Discussion

In this study, the ambient air of the exposed community had higher concentrations of PM_2.5_ and PM_10_. Furthermore, there was significant association between PM and incidence of respiratory symptoms and lung function for residents; and all symptoms studied were several folds higher compared to the control community. FEV1 and FVC were observed to be lower in residents living near the cement factory compared to those in the control community while the spirometric airflow limitation (FEV1/FVC ratio) was also lower for the exposed group compared to the control.

The 24-h averages of PM_10_ and PM_2.5_ levels were above the minimum recommended by WHO; on 21 days of the 42 days’ follow-up period PM levels were as high as 5 times the recommended levels. Although our study showed high PM levels, ranging from 3.6 to 168 μg/m^3^ and 0.4 to 54 μg/m^3^ for PM_10_ and PM_2.5_ respectively, similar studies have demonstrated much higher levels of PM in communities residing near cement factories. For instance, Kabir [24] reported an average concentration of 500 μg/m^3^ and 650 μg/m^3^ in two communities; Abdul et al. [22] found concentration levels ranging from 196.19 μg/m^3^ to 423.83 μg/m^3^ (particle size 0 to <150 μg) and Marcon et al. [25] reported average of 1208 μg/m^3^ of PM_10_ 24-h mean concentration over a period of 9 months. Furthermore, PM_10_ concentrations showed strong seasonal trends; the hot dry season had the highest (68.2 μg/m^3^) compared to cold (35.4 μg/m^3^) and rainy seasons (6.05 μg/m^3^). These findings are consistent with other studies [8,23] and may be attributed to changes in wind velocity, temperature, relative humidity, and precipitation magnitude and frequency [33]. Another factor could be that on certain days more PM emissions could have been released from the plant. Even slight variations in the emissions control could greatly impact the community-level PM concentrations on some days, as there are no other industrial activities nearby.

Evidence in literature shows that excessive exposure to PM, either acute or chronic effects (in a 24-h period or prolonged period), is associated with increased respiratory symptoms such as cough, phlegm, acute and chronic bronchitis, nasal irritation and reduced lung indices [14,15,34,35,36]. In this study, concentrations of PM were higher in the exposed community compared to control in all seasons. In this study, concentrations of PM were higher in the exposed community compared to control in all seasons, and PM_2.5_ in the exposed community accounted for a larger proportion of the PM_10_ that was measured, compared to control. Toxicological and epidemiological studies [37,38] suggest that PM_2.5_, since they are smaller and more likely to penetrate deeper into the lungs and blood streams unfiltered, could lead to respiratory and cardiovascular diseases. Our finding is cause for concern as participants from the exposed community are at risk of suffering from not only respiratory ill effects but also potential cardiovascular effects not investigated in this study.

Cough was the most reported respiratory symptom in both communities, although the incidence was higher in the exposed community than the control. Additionally, the chance that individuals without a cough transitioning to reporting a cough over time was three times higher in the exposed community compared to the control. Cough is the most basic response to airway irritation; nearly any type of irritation would induce cough compared to other symptoms. These findings are similar to other reports [8,25,39,40] and have also been demonstrated by epidemiological research in occupational settings [13,14,41]. Another symptom that was commonly reported in both communities was nasal irritation. In this study, PM_2.5_ and PM_10_ were significant determinants of both cough and phlegm controlling for area of residence.

There was lower performance on lung indices (FEV1 and FVC), reduced percentage of the predicted values for FEV1 and FVC, among the exposed community compared to the control; at baseline and subsequent seasons. Additionally, the mean lung indices for participants from the exposed community showed wider variations, compared to the control. Although the setting are different from other studies, our results are consistent with findings from studies in occupational settings [14,42]. This may be explained due to pre-existing effects of PM on the participants as most of the participants had lived in the area before the commencement of the study. The wider variation on the lung indices could further be explained by the variability of individual response to the atmospheric irritant. The individual response in-turn is dependent on factors such as the extent of lung damage already sustained, physiological adaptation and genetic make-up, and levels of exposure [43]. The reduction in the lung indices in this population may be suggestive of early obstructive lung disease such as chronic asthma, that participants may be suffering from but may not be aware of; literature shows that such diseases are either poorly diagnosed or under reported in most developing countries [44,45,46].

In this study, we found associations between PM and respiratory symptoms and lung indices. Assessment of PM and respiratory symptoms revealed that PM_2.5_ increased the odds of cough, phlegm production and nose irritation by 2.4%, 1.8% and 0.8% respectively. However, PM_2.5_ had a protective effect on wheeze. PM_10_ increased the odds of reporting phlegm production and nose irritation but the effect was delayed up to 3–5 days. Lung indices were lowered with increasing concentration of PM. Similar findings have been reported from other studies [26,47] on the association of ambient air pollution and respiratory ill health and also high levels of particulate matter in residents near industrial plants [23,25,48]. Both respiratory symptoms and reduced lung functions have been consistently associated with exposure to PM; duration and frequency of exposure tend to be determining factors [36,39,40,49,50]. Exposure to PM has been repeatedly associated with decreased FEV1 in human studies [32,42,51]. A single or short-time exposure to cement dust may not cause serious harm but exposure to cement dust of sufficient duration may cause serious irreversible health conditions [36]. Several other studies have reported associations between PM_10_ and acute effects such as increased daily mortality and increased rates of hospital admissions for exacerbation of respiratory disease [52,53]. Nkhama et al. reported that respiratory illnesses recorded at the only public health facility serving the exposed area was above the national prevalence rates of 136/1000 in 2013 [28].

The higher sensitivity of respiratory symptoms, compared to lung function, has been found in studies assessing effects of air pollution on respiratory health. Two possible mechanisms have been postulated; a biological effect of chronic exposure to low levels of air pollution without physiological changes or an increased perception of symptoms by people living near exposed areas [54]. The knowledge of levels of air pollution in Freedom compound are quite high; there has been wide media publication about the possible air pollution from the cement factory. Therefore, there is a possibility that residents from this community could have exaggerated the reported effects. However, it is also possible that PM from cement dust acts more acutely on lung function but the changes may be more transient than the occurrence of symptoms or may be more transient than the occurrence of symptoms present in vulnerable subjects.

In this study, there were no significant differences in major confounding variables such as demographic, length of stay in either exposed or control community, age, type of fuel used for cooking or lighting and gender.

The demographic and social characteristic of the two communities, save for gender, were comparable. The proportion of female participants was much higher in Freedom than Bauleni. At the time of enrolment, a high proportion of potential participants in Bauleni had relocated from the community. Most of the relocated were male therefore skewing the distribution of the sampling frame towards female gender. This may not be unexpected as literature shows that mobile populations tend to be males aged 16–29 [55]. Gender in this study thus was a potential confounder that needed controlling for in multivariable analyses.

Although this study adds to the evidence of associations of ambient air pollution with lung function in adults at very low levels, our findings should be interpreted with caution. Precise measurements require direct measurements of the pollutant that includes personal air monitoring and biological markers, however, in this study; only fixed community–level monitoring was performed to measure PM. This may not have captured fully the spatial and temporal heterogeneity of an individual’s personal exposure due to a combination of personal behaviors and micro-environmental sources. As a result, individual exposure estimates derived from ambient monitoring data may be subject to measurement error. It is also possible that pollutants, other than PM, could be responsible for the observed adverse health outcomes. Moreover, chemical characterization to ascertain the source apportionment was not conducted, making it impossible to conclude with certainty that the difference observed was due to cement dust. Therefore, future studies should comprise a component of chemical characterization in order to increase the certainty of the real cause. Understanding of the chemical constituents and sources of PM_2.5_ are warranted for designing effective emission control policies [54,56,57]. Further, non-participation of some subjects during follow up may bias observed associations or limit generalizability. However, several other elements of the study design strengthen our results. For instance, the daily repeated measures of both exposure and symptom outcomes across multiple seasons and the use of multivariable models allowed for adjustment of within-subject and between subject correlation and also accounted for temporal trends and other potential confounders. Additionally, the policy relevancy of our findings is strengthened by observation that even individuals who are seemingly healthy could be vulnerable to relatively low levels of PM exposure. Further, communities in the windward and downstream may be affected by PM. Future research should include conducting a study that would measure PM in the communities downstream and windward, including assessing chemical characterization in order to quantify sources of PM.

## 5. Conclusions

Findings from this study add to the body of knowledge that even seemingly healthy people are adversely affected due to exposure to PM at low levels. PM increased the likelihood of suffering from respiratory symptoms and lowered lung function indices. With increasing production and use of cement as has been witnessed in Zambia, effective public and environmental health policies that aim to reduce pollution levels for residents near cement industries could reduce the impact on respiratory health.

## Figures and Tables

**Table 1:** Baseline characteristics study population.

Characteristic	Freedom *n* = 63	Bauleni *n* = 55	
	*n* (%)	*n* (%)	*p*-Value
**Gender**			
Males *n* (%)	33 (52.4)	15 (27.3)	
Female *n* (%)	30 (47.6)	40 (72.7)	0.006
Employed Yes *n* (%)	46 (70.0)	38 (67.9)	0.062
Age (yrs) median(IQR)	30 (24–37)	33 (25–42)	0.173
Weight (kg) median (IQR)	63.0 (60.0–69.0)	61.0 (57–70.0)	0.312
Height (cm) median (IQR)	166 (161–171)	162 (158– 167)	0.004
BMI median (IQR)	25.2 (18.7–41.2)	24.8 (18.3–35.6)	0.297
Asthma	4 (6.3)	2 (3.6)	0.684 ^‡^
**Smoking status *n* (%)**			
Never	58 (92.0)	47 (85.4)	0.386
Former	2 (3.2)	5 (9.1)	
Current	3 (4.8 )	3 (5.5)	
**Source of energy *n* (%)**			
Lighting			
Electricity	51 (85.0)	46 (85.2)	
Candle	9 (15.0)	8 (14.8)	0.978
**Cooking**			
Electricity	33 (55.9)	31 (59.6)	
Charcoal	26 (44.1)	21 (40.4)	0.695

% percentage; kg kilograms; IQR interquartile range; yrs years; ^‡^ Fisher’s exact.

**Table 2:** Mean concentration (μg/m^3^) of particulate matter and meteorological variables for the exposed and control communities.

Variable	Location	Cold Dry	Hot Dry	Warm Rainy	Total
PM_2.5_	Freedom	9.15 (11.58)	24.93 (22.06)	2.39 (2.67)	10.21 (15.55)
x (sd)	Bauleni	6.03 (5.25)	6.89 (10.91)	1.69 (1.28)	5.87 (5.87)
	^†^	0.001	0.025	0.001	0.001
PM_10_	Freedom	31.40 (43.38)	68.28 (53.92)	7.03 (6.32)	36.96 (47.76)
x, (sd)	Bauleni	7.66 (6.33)	8.82 (6.32)	2.26 (0.71)	6.67 (7.70)
	^†^	0.001	0.001	0.001	0.001
Temp.	Freedom	20.72(1.60)	30.22 (1.62)	23.27 (3.94)	24.64 (4.79)
°C, (sd)	Bauleni	20.72 (1.60)	30.29 (1.62)	22.16 (3.24)	24.35 (4.79)
	^†^	1.000	0.004	0.020	0.040
RH	Freedom	44.79 (5.02)	70.47 (10.57)	71.87 (8.09)	61.86 (15.01)
%, (sd)	Bauleni	44.79 (5.01)	70.46 (10.55)	72.30 (7.90)	62.05 (15.05)
	^†^	1.000	0.200	0.505	0.073

^†^ *p* value for the difference between communities; Temp temperature; RH relative humidity; sd standard deviation.

**Table 3:** Symptoms incidence rate (per 100 person-days) by season and community; and transition probabilities.

	Cold		Hot Dry		Warm Rain		Overall		Transition Probability	
	Bauleni	Freedom	Bauleni	Freedom	Bauleni	Freedom	Bauleni	Freedom	Bauleni	Freedom
	*n* = 726	*n* = 821	*n* = 768	*n* = 889	*n* = 699	*n* = 850	*n* = 2193	*n* = 2560		
Symptom	*n* (%)	*n* (%)	*n* (%)	*n* (%)	*n* (%)	*n* (%)				
Cough	94 (12.9)	431 (52.5)	122 (15.9)	668 (75.1)	87 (12.4)	87 (10.4)	303 (13.8)	1186 (46.3)	8.83	27.6
Phlegm	26 (3.6)	386 (47.0)	122 (15.9)	584 (65.7)	16 (9.0)	86 (10.1)	211 (9.6)	1056 (41.2)	6.20	28.23
Nose	72 (9.9)	410 (49.9)	145 (18.9)	653 (73.4)	57 (8.2)	191 (22.5)	274 (12.5)	1254 (49.0)	1.70	10.07
Wheeze	9 (1.2)	90 (11.0)	40 (5.2)	201 (22.6)	37 (5.3)	65 (7.6)	86 (3.9)	356 (13.9)	8.74	36.90

**Table 4:** Percent predicted lung function variations between communities and season.

	Season						Overall	
	Cold		Hot		Warm Rainy			
	Freedom	Bauleni	Freedom	Bauleni	Freedom	Bauleni	Freedom	Bauleni
FEV1								
Mean (sd)	91.56 (22.45)	96.54 (14.53)	89.67 (19.30)	94.01 (14.21)	91.20 (20.53)	99.39 (14.37)	90.74 (20.62)	96.59 (14.53)
FVC								
Mean (sd)	94.25 (22.37)	97.77 (15.10)	91.13 (19.65)	94.61 (15.98)	93.11 (21.63)	100.97 (17.31)	93.69 (21.16)	97.70 (16.44)
FEV1/FVC ratio								
Mean (sd)	0.81 (0.10)	0.83 (0.09)	0.82 (0.10)	0.84 (0.09)	0.83 (0.86)	0.83 (0.88)	0.82 (0.10)	0.83 (0.09)

sd = standard deviation.

**Table 5:** Adjusted OR estimates of association between PM_2.5_ and PM_10_ and respiratory symptoms among participants from Freedom and Bauleni communities.

	Cough	Phlegm	Wheeze	Nose Irritation
	OR (95% CI)	OR (95% CI)	OR (95% CI)	OR (95% CI)
Exposure to PM_2.5_				
PM_2.5_ lag1	1.02 (1.01, 1.04) *	1.02 (1.01, 1.03) *	0.99 (0.98, 1.00) *	1.01 (1.01, 1.02) *
Exposure to PM_10_				
PM_10_ lag1	1.00 (0.99, 1.00)	0.99 (0.99, 1.00) *	1.00 (0.98, 1.00)	0.99 (0.99, 1.00) *
PM_10_ lag3	-	-	-	1.00 (1.00, 1.01) *
PM_10_ lag5	-	1.00 (1.00, 1.01) *	-	1.00 (1.00, 1.01) *

OR-odds ratio; CI-confidence level by 10 μg/m^3^ in PM_2.5_ and PM_10_, *- *p* value < 0.005. Controlled for time, smoking status, season, asthma. Lag 1 to 7 was investigated for both PM_2.5_ and PM_10_. Only lags effects that were statistically significant for any given symptoms are shown in the table.

**Table 6:** Adjusted estimates of associations between PM concentrations and lung function.

Single Pollutant	Freedom	*p* Value	Bauleni	*p* Value
	Coefficient (95% CI)		Coefficient (95% CI)	
PM_2.5_ FEV1				
PM_2.5_ lag1	0.29 (−0.25, 0.83)	0.294	0.30 (−0.45, −1.04)	0.435
PM_2.5_ lag2	0.23 (−0.31, −0.77)	0.402	0.65 (0.09, 1.39)	0.085
PM_2.5_ lag3	0.08 (−0.49, −0.65)	0.971	−0.16 (0.87, 0.55)	0.664
PM_2.5_ lag4	0.12 (−0.42, −0.67)	0.657	0.31 (−0.05, 1.07)	0.424
PM_2.5_ lag5	−0.07 (−0.63, −0.40)	0.800	0.58 (−0.15,−1.31)	0.811
PM_2.5_ FVC				
PM_2.5_ lag1	0.15 (−0.44, −0.73)	0.627	0.15 (−0.71, 1.02)	0.726
PM_2.5_ lag2	0.08 (−0.49, −0.66)	0.775	0.76 (−0.12, 1.62)	0.086
PM_2.5_ lag3	0.17 (0.44 ,0.79)	0.585	0.23 (−0.60, 1.07)	0.582
PM_2.5_ lag4	0.22 (0.35, 0.79)	0.458	0.66 (−0.21, 1.53)	0.136
PM_2.5_ lag5	−0.08 (−0.67, −0.51)	0.787	0.658 (0.18, 1.48)	0.126
PM_10_ FEV1				
PM_10_ lag1	−0.07 (−0.25, −0.11)	0.459	−0.37 (−1.25, 0.52)	0.416
PM_10_ lag2	−0.06 (−0.23, −0.11)	0.488	0.87 (0.01, 1.65)	0.028 ^a^
PM_10_ lag3	−0.15 (−0.34, −0.30)	0.098	0.14 (0.90, 20.16)	0.728
PM_10_ lag4	0.08 (−0.10, −0.26)	0.365	0.22 (0.99, 0.51)	0.560
PM_10_ lag5	−0.15 (−0.33, −0.03)	0.092	−0.30 (−1.03, 0.43)	0.424
PM_10_ FVC				
PM_10_ lag1	0.01 (−0.18, −0.21)	0.910	−0.74 (−1.76, 0.27)	0.152
PM_10_ lag2	−0.06 (−0.24, −0.12)	0.514	0.78 (−0.11, 1.68)	0.086 ^b^
PM_10_ lag3	−0.13 (−0.33, −0.06)	0.173	−0.04 (−0.94, −0.86)	0.935
PM_10_ lag4	0.79 (−0.07, −1.67)	0.073	0.04 (−0.15, −0.22)	0.704
PM_10_ lag5	−0.26 (−0.45, −0.08)	0.006	0.13 (0.96, 0.70)	0.751

Controlled for age, gender, weight, height, smoking status, asthma, occupation, temperature, humid, lighting fuel, cooking fuel, season, cook outside; ^a^ statistically significant (*p* value < 0.05);^b^ marginally significant (*p* value).

## Abbreviations

The following abbreviations are used in this manuscript:

PM	Particulate matter
CL	Confidence level
OR	Odds ratio
WHO	World Health Organisation
MoH	Ministry of Health
CHW	Community Health Workers
USEPA	United States Environmental Protection Agency
NIEHS	National Institute of Environmental Health Sciences
